# Hypoxia induces TFE3 expression in head and neck squamous cell carcinoma

**DOI:** 10.18632/oncotarget.7309

**Published:** 2016-02-10

**Authors:** Zhi-Jun Sun, Guang-Tao Yu, Cong-Fa Huang, Lin-Lin Bu, Jian-Feng Liu, Si-Rui Ma, Wen-Feng Zhang, Bing Liu, Lu Zhang

**Affiliations:** ^1^ The State Key Laboratory Breeding Base of Basic Science of Stomatology & Key Laboratory of Oral Biomedicine Ministry of Education, Wuhan, China; ^2^ Department of Oral Maxillofacial-Head Neck Oncology, School and Hospital of Stomatology, Wuhan University, Wuhan, China

**Keywords:** hypoxia, head and neck cancer, TFE3, chemotherapy

## Abstract

To assess the role of transcription factor μE3 (TFE3) in the tumorigenesis of head and neck squamous cell carcinoma (HNSCC), human HNSCC tissue arrays were investigated for TFE3 expression. Human HNSCC tissues with neoadjuvant inductive chemotherapey (docetaxel, cisplatin and fluorouracil, TPF) and mice HNSCC tissues from transgenic mice model were evaluated for TFE3 expression and the hypoxia pathway. The roles of EGF/EGFR mediated hypoxia in TFE3 nuclear expression were analyzed *in vitro* and *in vivo*. TFE3 expression was higher in human HNSCC tissues compared with that in normal oral mucosa. Moreover, high TFE3 expression was related to HIF-1α, PAI-1, and EGFR, which demonstrated the activation of the hypoxia pathway in HNSCC tissues. Furthermore, elevated TFE3 expression was observed in HNSCC after cisplatin-based chemotherapy, and high TFE3 expression may indicate poor response to TPF inductive chemotherapy. Furthermore, similar changes with increased TFE3 were observed in HNSCC of the transgenic mouse HNSCC model. Hypoxic culture in the human HNSCC cell line increased TFE3 expression, which promoted cell survival under hypoxia. EGFR inhibiton by cetuximab could attenuate hypoxia-induced TFE3 in the HNSCC cell line and transgenic mouse HNSCC model. These findings indicated that TFE3 was an important hypoxia-induced transcriptional factor in HNSCC. TFE3 could be regarded as a durgable therapeutic oncotarget by EGFR inhibition.

## INTRODUCTION

Head and neck squamous cell carcinoma (HNSCC) is the sixth most common cancer in humans, with about 450,000 newly diagnosed cases worldwide every year [1]. HNSCC causes significant morbidity and mortality, with a five-year survival rate of <50%, and overall survival has remained relatively unchanged for the past three decades [2]. The molecular mechanism of dysregulation in HNSCC progression that involves the sequential acquisition of genetic and epigenetic alterations is widely unknown [3, 4].

The high levels of acidity and hypoxia in HNSCC cause low response to chemotherapy [5]. Hypoxia inducible factor (HIF) plays a key role in tumor, and it is correlated with short disease-free survival by upregulating hypoxia-related factors, such as transforming growth factor-β (TGF-β) and plasminogen activator inhibitor type-1 (PAI-1), which play an important role in antiapoptotic and angiogenic factors and cancer stem cell initiation [5]. Cellular processes affected by the TGF-β pathway include regulation of the differentiation and inhibition of epithelial cell proliferation and apoptosis [6, 7]. TGF-β ligands exert their activity by binding to a family of trans-membrane serine/threonine kinase receptors. TGF-β binding to these receptors initiates a signal cascade, in which the Smad proteins are the primary signal transducers. PAI-1 is a multifunctional protein best known for its role as an inhibitor of urokinase-type plasminogen activator (uPA), which is related to both hypoxia and the TGF-β pathway [8]. Despite its uPA-inhibiting function, PAI-1 has been demostrated by numerous clinical studies to have a strong correlation with poor HNSCC prognosis[9]. This paradoxical finding may be explained by further biological functions of PAI-1 in promoting migration and angiogenesis and in inhibiting apoptosis of tumor cells [10, 11]. Emerging basic and clinical findings indicated that epidermal growth factor receptor (EGFR)-mediated aberrant signaling transduction is crucial in HNSCC tumorigenesis and progression [12]. EGFR has been observed in 70% to 100% of all HNSCC lesions [12]. Cetuximab is a chimeric IgG1 monoclonal antibody that is currently licensed for the treatment of HNSCC patients[13]. Our previous study suggeted that inhibition of EGFR may reduce HIF-1α in a preclinical mouse HNSCC model[14].

Transcription factor μE3 (TFE3) is a member of the microphthalmia-TFE (MiTF) basic helix-loop-helix leucine zipper transcription factor subfamily [15, 16], which is known as an important co-activator of TGF-β transcription and regarded as a potential oncogenic transcription factor [17–19]. Very few molecular mechanisms have established TFE3 as a gene that can uncouple cell cycle progression from Rb phosphorylation and E2F3 and identify a functional role for TFE3 in regulating proliferation [17], cell survival, oxidative stress, lysosomal biogenesis, and autophagy (see review [20]). However, these studies on TFE3 have been mostly limited to genetic and biochemical studies. For human diseases, translocations involve TFE3 with several fusion gene partners, including *PRCC*, *NONO*, *SFPQ*, *CLTC*, and *ASPL*, in certain pediatric renal carcinomas and alveolar soft part sarcoma [21–24]. TFE3 over-expression was also found in parts of perivascular epithelioid cell tumors[20]. Nevertheless, reports on TFE3 expression in other solid tumors or it's correlation with hypoxia are relatively rare.

These observations prompted us to examine TFE3, which may play an important role in both TGF-β and the hypoxia pathway. In the present study, we found that TFE3 activation was a widespread event in spontaneously developed mice HNSCC and human HNSCC. This event was related to the hypoxia pathway.

## RESULTS

### Expression of TFE3 in human HNSCC from data mining on the ONCOMINE database

To determine whether TFE3 expression is associated with HNSCC, the ONCOMINE cancer microarray database was used [25] to study TFE3 gene expression in human HNSCC and their normal tissue counterparts. In Poage's dataset, DNA copy number analysis was independently performed on HNSCC. Fig. S1A showed amplification of the HNSCC copy number. In Cromer's dataset, mRNA expression analysis was independently performed on TFE3, and mRNA of *TFE3* in HNSCC was enhanced compared with that in normal tissues (Fig. S1B). In a previous study, Garnett et al. also indicated that TFE3 mRNA is abnormally accumulated in acadesine-resistant HNSCC cells compared with acadesine-sensitive HNSCC cells [26] (Fig. S1C). Furthermore, mRNA gene expression levels between HNSCC and normal oral tissues were systematically compared among five datasets from the ONCOMINE database. The results indicated that increased TFE3 expression was significantly associated with head and neck cancer compared with the normal counterpart (*P* = 0.032) (Figs. S1D and S1E). Based on the results above, we hypothesized that TFE3 is hypernomic activation in HNSCC. Therefore, further validation of TFE3 using immunohistochemistry in a cohort of HNSCC samples should be further exploited.

### Increased levels of TFE3, HIF-1α, PAI-1, and EGFR in human HNSCC tissue

**Figure 1:**
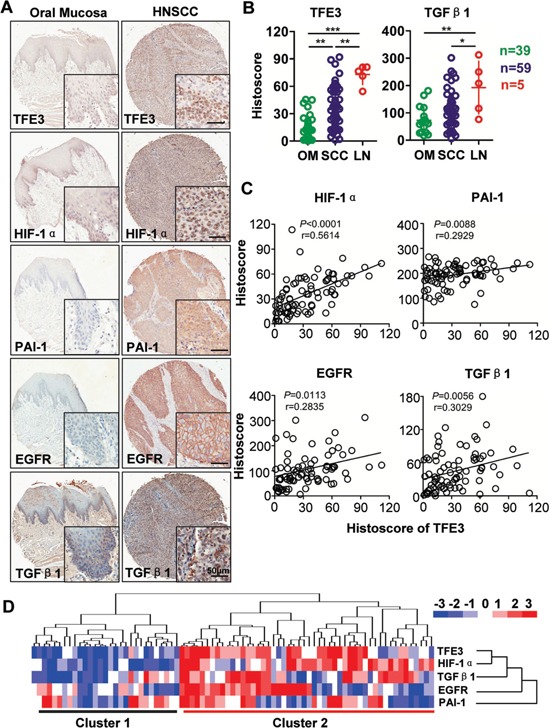
Increased TFE3 correlated with hypoxic factor in HNSCC **A.** Representative immunohistochemistry staining of TFE3, HIF-1α, PAI-1, EGFR and TGF-β1 in oral mucosa as well as in HNSCC tissue (Scale bars =50μm). **B.** Quantification of the histoscore of TFE3 and TGF-β1 in normal oral mucosa (OM, n=39), HNSCC (n=59) and lymph node metastasis (LN, n=5). (Graph Pad Prism 5, One way ANOVA with post-Tukey statistic), **,*P* <0.01; ***,*P* <0.001. **C.** Correlation and linear regression between the expression of TFE3 with HIF-1α, EGFR, PAI-1 and TGF-β1in human normal mucosa and HNSCC tissue (quantification including normal mucosa and HNSCC). **D.** Hierarchical clustering presents the protein expression correlation of TFE3, HIF-1α, EGFR, PAI-1 and TGF-β1 in human HNSCC tissue array, which exhibits the high expression of TFE3, HIF-1α, EGFR, PAI-1 and TGF-β1 in HNSCC (most in cluster 2) as compared with normal mocosa (most in cluster 1).

To assess the expression levels of TFE3, HIF-1α, PAI-1, and EGFR, tumor sections from human tissue arrays for HNSCC (*n* = 59) were stained with antibodies against TFE3, HIF-1α, PAI-1, and EGFR compared with oral mucosa (*n* = 39) and lymph node metastasis (n=5). Representative figures of TFE3, HIF-1α, PAI-1, and EGFR immunostaining are shown in Fig. 1A. Levels of TFE3 (*P* < 0.01), HIF-1α (*P* < 0.01), PAI-1 (*P* < 0.01), and EGFR (*P*<0.01) during immunostaining exhibit light stains in the nuclear area or cytoplasm of oral mucosa but are significantly increased in HNSCC cores (Fig. 1A and S2). TFE3 nuclear expression was also found to be even higher in lymph node metastasis (*n* = 5, *P* < 0.01, Fig. 1B). TFE3 expression was increased in high-grade HNSCC and large tumor size, but the difference was not statistically significant (Fig. S2A). Interestingly, PAI-1 expression significantly increased (*P* < 0.05) in poorly differentiated HNSCC samples compared with well-differentiated HNSCCs (Grade III vs. Grade I; Grade IV vs. Grade I; Grade IV vs. Grad II, *P*<0.05 respectively, Fig. S2B). In addition, PAI-1 expression increased in large-size HNSCC (T3 or T4) as compared with that in small-size HNSCC (T1, *P*<0.05, Fig. S2B).

To elucidate the potential association between TFE3 expression and hypoxia related factors in human HNSCCs, we used the Spearman rank correlation coefficient test and linear tendency test to evaluate the histoscore of immunostaining. TFE3 expression was positively correlated with higher expression of HIF-1α (*P* < 0.0001, r = 0.5614), EGFR (*P* = 0.0056, r = 0.3090), PAI-1 (*P* = 0.0088, r = 0.2929), and TGF-β1 (*P* = 0.0113, r = 0.2835). Quantification included HNSCC tissue and normal mucosa (Fig. 1C). Hierarchical clustering analysis demonstrated that HIF-1α expression was notably closer to TFE3 expression (Fig. 1D). These data suggested that increased TFE3 expression was associated with increased levels of HIF-1α, EGFR, PAI-1, and TGF-β1 in human HNSCCs.

### Cisplatin-based chemotherapy treatment induced TFE3 expression, correlating with hypoxia in human HNSCC

**Figure 2:**
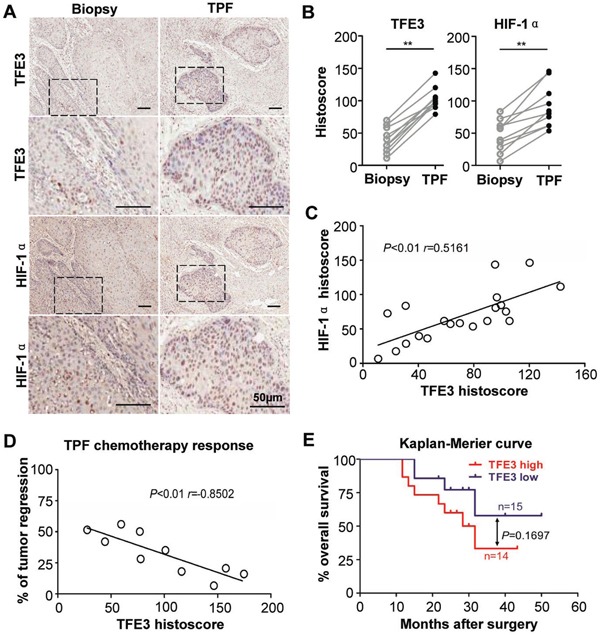
Increased TFE3 correlated with hypoxia in cisplatin- based chemotherapy **A.** Representative immunohistochemical staining of TFE3, HIF-1α in same HNSCC paitent biopsy or surgical specimen after 2 round inductive combined cisplatin, docetaxel, and fluorouracil (TPF) chemotherapy. **B.** The expression level of TFE3, HIF-1α after TPF chemotherapy was significantly higher than original HNSCC (paired t test, *P*<0.001). **C.** The expression of TFE3 was correlated with HIF-1α in TPF chemotherapy HNSCC(*P*<0.01). **D.** Expression of orginal TFE3 in biopsy negative correlated with regression after TPF chemotherapy (*P*<0.01). **E.** The prognosis value of TFE3 in HNSCC was analyzed by Kaplan-Meier, log-Rank analysis reveal the difference was not significant (*P*>0.05). Scale bars =50μm.

To analyze underlying cellular processes affected by sequential neoadjuvant (cisplatin, docetaxel, and fluorouracil, TPF) chemotherapy, immunohistochemistry was performed in an HNSCC specimen using inductive TPF chemotherapy and paired biopsy in the same patient. Results showed that the epithelial island regressed after TPF chemotherapy (Fig. 2A), but the expression levels of TFE3 and HIF-1α evidently increased in the residual tumor island when compared with paired biopsy (*P* < 0.01, Fig. 2B). TFE3 expression was correlated with HIF-1α expression in TPF chemotherapy sample (*P*<0.01, *r*=0.5161, Fig. 2C). Remarkably, increased TFE3 expression indicated a poor response to TPF chemotherapy (*P* < 0.01, *r* = −0.8502, Fig. 2D). To explore the prognostic value of TFE3 in HNSCC with inductive TPF chemotherapy, Kaplan–Meier analysis was conducted. As shown in Fig. 2E, TFE3 expression may indicate a rather poor prognosis of HNSCC patients, whereas log-rank analysis indicated that the cumulative overall survival rate by TFE3 (*P* = 0.1697) expression did not reach statistical significance. To further validate this finding, human esophagus cell carcinoma tissue array was used (*n* = 93 ESCC with 79 paired esophagus mucosa), which contained 60–72 month follow-up information in 85 patients. As indicated in Fig. S3, high TFE3 expression was distinct from low TFE3 expression among ESCC patients, which may have poorer overall survival. This finding was also not statistically significant (*P* = 0.1291). Therefore, these data indicated that TFE3 expression was correlated with cisplatin-based chemotherapy in HNSCC, but had limited prognostic indication.

### EGFR inhibition attenuates hypoxia-induced TFE3 and inhibits cell proliferation in human HNSCC cell lines

**Figure 3:**
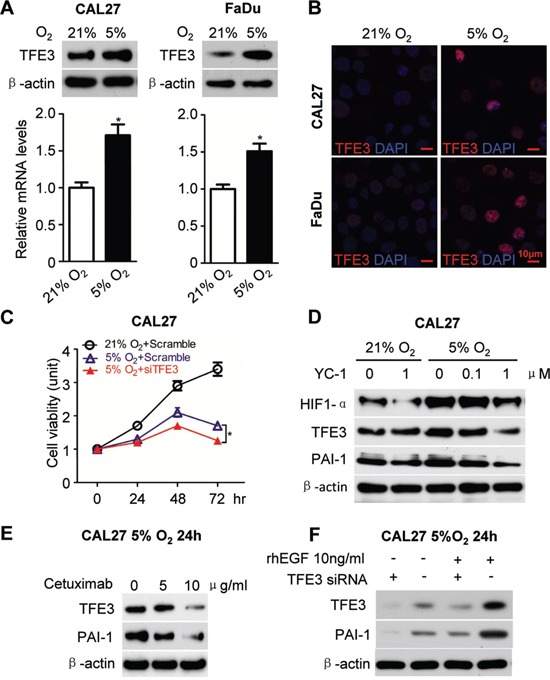
EGFR inhibition attenuated hypoxia induced TFE3 expression **A.** Increased mRNA level as well as protein level for TFE3 medium hypoxia culture condition (5% O_2_, 24h) in the HNSCC cell line CAL27 (left panel) and FaDu (right panel) as compared with normoxia. **B.** Immunofluroscence for increase of TFE3 nuclear expression by hypoxia culture condition (5% O_2_, 24h) in CAL27 (upper) and FaDu (lower) cell lines. **C.** *In vitro* knock down of TFE3 by siRNA significant reduce cell viability of CAL27 in hypoxia culture condition at 48 and 72 h (*P*<0.05). **D.** Western blot analysis of HIF-1α, TFE3, PAI-1 after hypoxia inhibitor compound YC-1 treatment at a dose dependent manner in hypoxia culture and normoxia culture condition. **E.** Western blot analysis of TFE3 and PAI-1 in the HNSCC cell line CAL27 after Cetuximab treatment in a dose dependent manner in hypoxia culture condition at 24h. **F.** Western blot analysis of TFE3 and PAI-1 in the HNSCC cell line CAL27 with recombination human EGF (10 ng/ml) and TFE3 siRNA treated in hypoxia culture condition at 24h. Quantification is performed using Image J by pixel analysis of band by normalized of β-actin as a loading control. The experiments were repeated twice with triplicate.

Subsequently, the effect of hypoxia on TFE3 expression was determined. Hypoxic cultures using Anoxomat chambers were utilized. Interestingly, an increase in TFE3 expression was observed in hypoxic culture conditions in both the mRNA and protein levels (5% O_2_, 24 h, Fig. 3A). The result was quite consistent in the two HNSCC cell lines CAL27 and FaDu. Immunofluorescence also indicated an increase in TFE3 nuclear expression caused by hypoxic culture conditions in CAL27 and FaDu (Fig. 3B). For the functional assay, TFE3 was knocked down using siRNA. We chose two predesigned siRNA sequences to knock down TFE3. Both TFE3 siRNA sequences could knock down TFE3 by more than 80% at a final concentration of 5 nM at both the mRNA and protein levels (Figs. S4A and S4B). TFE3_2 was selected for subsequent experimentation. As shown in Fig. 3C, a significant reduction in TFE3 siRNA decreased cell viability of CAL27 in hypoxic culture condition at 48 and 72 h (*P* < 0.05 in 72 h). This finding indicated that knocked down TFE3 may reduce cell viability in hypoxic conditions. To prove that the increase in TFE3 was related to HIF-1α, a hypoxia inhibitor YC-1 was used in nornomia and hypoxic conditions. Interestingly, YC-1 treatment reduced the effect on HIF-1α, TFE3, and PAI-1 expression in a dose-dependent manner during hypoxia, whereas minimal effects were observed under normoxia (Fig. 3D). Our previous study showed that EGFR inhibition by cetuximab significantly reduces nuclear translocation of HIF1α in a human HNSCC cell line *in vitro* and *in vivo*. Cetuximab treatment reduced TFE3 and PAI-1 in a dose-dependent manner, as indicated by Western blot analysis results (Fig. 3E). Moreover, exogenous applied recombinant human EGF (10 ng/ml) increased TFE3 and PAI-1 expression in the CAL27 cell line, which was attenuated by TFE3 knock down (Fig. 3F). The experiments mentioned above were repeatable in the FaDu cell line (Figs. S4C–F).

### TFE3 upregulation is correlated with HIF-1α and PAI-1 in *Tgfbr1/Pten* 2cKO mice bearing spontaneously developed HNSCC tumors

**Figure 4:**
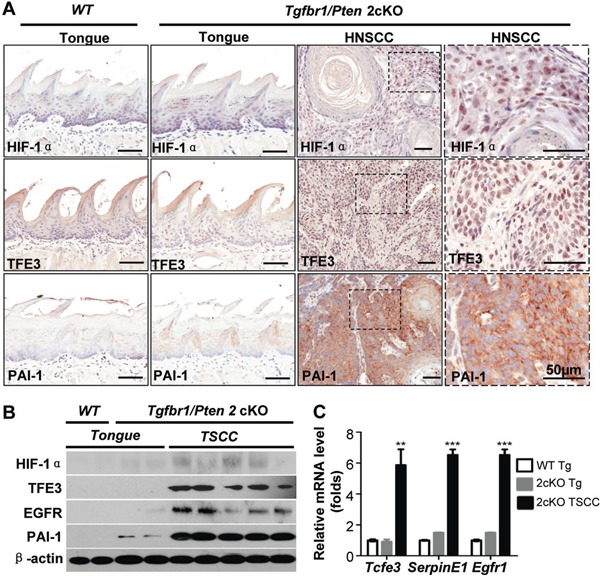
TFE3 upregulation is correlated with HIF-1α and PAI-1 in Tgfbr1/Pten 2cKO mice bearing spontaneously developed HNSCC tumors **A.** Immunohistochemical staining shows increased expression of TFE3, HIF-1α and PAI-1 in *Tgfbr1/Pten* 2cKO mouse HNSCC (high magnification in the right panel), as compared with *Tgfbr1/Pten* 2cKO mouse mucosa and *Tgfbr1^flox/flox^*/Pten*^flox/flox^* mucosa (Scale Bars=50μm). **B.** Western blot shows a significant increase in TFE3, HIF-1α, PAI-1 and EGFR in *Tgfbr1*cKO mice HNSCC and *Tgfbr1/Pten* 2cKO mice HNSCC compared with counterpart tongue mucosa (Tg, tongue; TSCC, tongue squamous cell carcinoma). **C.** Quantitative Real-time PCR revealed the mRNA level of *Tcfe3* (the gene name for TFE3), *Serpin E1* (the gene name for PAI-1) *and Egfr1* (the gene name for EGFR) in 2cKO HNSCC as compare to *Tgfbr1/Pten* 2cKO tongue and wide type tongue (One way ANOVA, Mean±SEM, **, *P*<0.01,***, *P*<0.001).

HNSCC tumorigenesis by deletion of *Pten* (*Pten* cKO mice), *Tgfbr1* (*Pten* cKO mice with DMBA), and combined deletion of *Tgfbr1* and *Pten* (*Tgfbr1/Pten* 2cKO mice) has already been reported [27]. To investigate whether TFE3 activation occurs in mice HNSCC tumors, our analysis revealed intense staining in TFE3 in *Tgfbr1/Pten* 2cKO HNSCC. HIF-1α, TFE3, EGFR, and PAI-1 expression increased in mice HNSCC compared with that in the oral mucosa, as indicated by immunohistochemistry (Fig. 4A with quantification in Fig. S5) and Western blot analysis (Fig. 4B). Increased mRNA levels of *Tcfe3, Serpin E1*, and *Egfr1* were also observed in 2cKO HNSCC compared with *Tgfbr1/Pten* 2cKO tongue and wide-type tongue using real-time PCR (Fig. 4C).

### Inhibiting EGFR by cetuximab decreases HIF1α.-induced TFE3 and PAI-1 expression in *Tgfbr1/Pten* 2cKO mice

**Figure 5:**
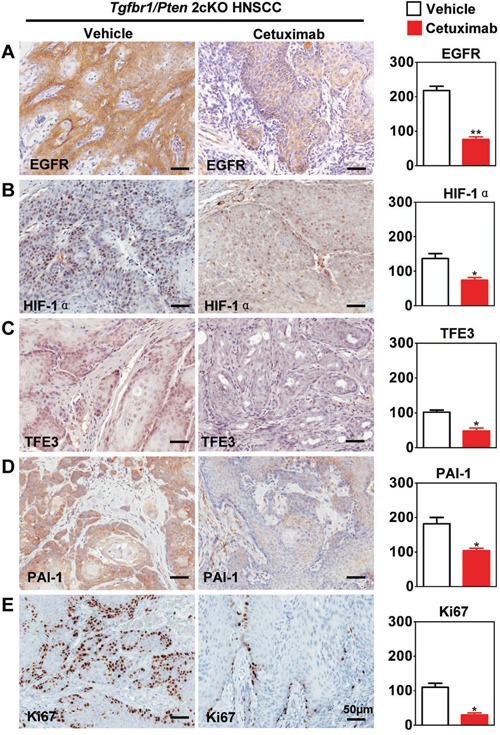
Targeting hypoxia by cetuximab decrease TFE3 in *Tgfbr1/Pten* 2cKO mice Immunohistochemical staining of HIF-1α, TFE3, PAI-1, EGFR and proliferating marker Ki67 in *Tgfbr1/Pten* conditional knock out mice after cetuximab treatment and with quantitative analysis. The expression of HIF-1α, TFE3, PAI-1, EGFR and Ki67 were was significantly reduced in the cetuximab treatment group than the vehicle group (t test, *, *P*<0.05, **, *P*<0.01). Scale bars =50μm.

To analyze the effect of hypoxia on TFE3 *in vivo*, cetuximab were used in *Tgfbr1/Pten* 2cKO, which confirmed a decrease in hypoxia and decrease in HIF-1α [14]. Consistent with our previous study, cetuximab treatment significantly decreased EGFR (*P* < 0.01, Fig. 5A) and HIF-1α (*P* < 0.01, Fig. 5B) expression. Further, we found that cetuximab treatment also decreased TFE3 nuclear expression (*P* < 0.05, Fig. 5C) and PAI-1 (*P* < 0.05, Fig. 5D) cytoplasmic expression. Additionally, we detected down-regulation Ki67 after cetuximab treatment (*P* < 0.05, Fig. 5E). These findings suggested that EGFR inhibition by cetuximab may attenuate hypoxia-induced TFE3 expression. TFE3 may play a role in maintaining HNSCC cell viability during hypoxia., as well as putative proliferation.

## DISCUSSION

A newly gained molecular understanding on HNSCC initiation and progression may soon present the opportunity to develop novel drug targets and therapeutic approaches. In the present paper, we report a significant increase in TFE3 expression in human HNSCC tissues based on *in silico* bioinformatics analysis and tissue microarray immunohistochemistry. Over-expressed TFE3 in HNSCC may be related to hypoxia, as indicated by the significant correlation of HIF-1α, TGF-β1, and PAI-1. Furthermore, increased TFE3 levels in HNSCC may be correlated with cisplatin-based chemotherapy resistance, but have a limited role in prognostic prediction. Mechanistically, we confirmed that hypoxia increased TFE3 expression, which may play an important role in maintaining cell viability. Moreover, EGFR inhibition by cetuximab was found to possibly be an effective strategy of TFE3 targets *in vitro* and *in vivo*.

TFE3 over-expression in HNSCC is related to hypoxia. Hypoxia is an important feature of cancer biology: it leads to resistance to radiotherapy and anticancer chemotherapy. During hypoxia, multiple cellular pathways are activated [28]. Given that increased TFE3 in HNSCC is related to the HIF pathway, TFE3 expression increased in a time-dependent manner under hypoxia [29], which was inhibited when treated with a hypoxia inhibitor [30]. Furthermore, using a genetically engineered mouse model that spontaneously developed visible HNSCC tumors on the tongue and facial region by conditional deletion of *Pten* and *Tgfbr1* [31], TFE3 activation was shown as a significant molecular event in HNSCC. Previous data suggested that tumorigenesis of *Tgfbr1/Pten 2cKO* mice has evident papilloma stages, and high molecular expression of miR-135b regulates HIF-1α [32]. Our data were consistent with the report that tumor cells in alveolar soft part sarcoma, a malignancy with known TFE3 fusion expression, exhibit TGF-β1-dependent (Fig. S6), hypoxia-regulated cytoglobin stellate cell activation association protein (cytg/STAP) [30]. The proliferation of TFE3-regulated cells during hypoxia may directly bind with E2F3 (Fig. S6), an important transcription factor of the Rb pathway [33], which warrants further investigation. However, the present study did not show evidence of whether TFE3 gene translocation is associated with HNSCC, which requires further studies.

Interestingly, TFE3 expression was correlated with the cisplatin-based chemotherapy response in HNSCC. Dysregulation of MiT family members has been evaluated for prognostic and predictive significance. In the melanocytic lineage, MITF may be a determinant of survival after irradiation, but its association with chemotherapy resistance is controversial [34, 35]. TFE3- and TFEB-associated cancers are generally considered difficult to treat through chemotherapy or irradiation, which may be related to the activation of survival factors, similar to MiTF [20]. Our findings suggested the potential predictive role of TFE3 in cisplatin-based chemotherapy of HNSCC. Admittedly, a relatively small number of clinical samples clearly limited our study. Thus, these marker genes must be re-examined in clinical studies with a larger number of patient samples. An interesting study reported 39 cases of genetically confirmed translocation renal cell carcinoma patients; six (15%) of these patients had a history of receiving chemotherapy at childhood, which indicated that chemotherapy may increase the possibility of TFE3 translocation [36]. TFE3 amplification is reportedly correlated with poor prognosis of melanoma [35, 37]. In the present study, TFE3 expression may indicate a rather poor prognosis of HNSCC patients, whereas log-rank analysis indicated that the cumulative rate of the patients with TFE3 expression did not reach statistical significance.

TFE3 may be a drugable target by EGFR inhibition. Accumulated evidence showed that the persistent over-expression and activation of EGFR have emerged as putative drug targets for HNSCC treatment in preclinical and clinical investigations [12, 38, 39]. The clinical application of EGFR inhibitor in HNSCC therapy is a revolutionary event [12]. Recent reports confirmed that cetuximab may enhance radiosensitization by decreasing hypoxia in cancer [40–42]. Our previous and present findings also suggested a significant activation of the EGFR/HIF-1α pathways, in addition to over-expression of VEGFA in this mouse model [14, 32]. Moreover, EGFR inhibition led to HIF-1α inhibition [14]. We confirmed that EGFR inhibition attenuated hypoxia-induced TFE3 expression and decreased cell viability under hypoxia. Alternatively, the findings provide a novel rationale to target hypoxia-induced TFE3 through EGFR inhibition.

Taken together, this study demonstrated that hypoxia-induced TFE3 over-expression may regulate cell viability. TFE3 may also be a drugable oncotarget by currently approved EGFR inhibitors for HNSCC patients.

## MATERIALS AND METHODS

### Cell culture, hypoxia culture, cell viability and RNA interference

Genotyping confirmed HNSCC cell line CAL27 and FaDu (ATCC, Manassas, VA) was maintained in Dulbecco's modified Eagle's medium (DMEM), 10% fetal bovine serum, at 5% CO_2_ and 37°C in a humidified incubator as previously described [27, 31, 43]. When cells were grown to 70% confluence, they were suspended in a-minimal essential medium with 10% FBS, and further cultured for indicated time (0–24 h) in Anoxomat chambers (Mart Microbiology, Lichtenvoorde, the Netherlands) with appropriate oxygen concentrations for hypoxia (5% O_2_) or normoxia (21% O_2_) as previous described [44]. For functional analysis, non-targeting negative control siRNA (Qiagen, Valencia, CA), TFE3 siRNA (Hs_TFE3_2 FlexiTube siRNA and Hs_TFE3_2 FlexiTube siRNA), were transfected into the appropriate cells using Hiperfect transfection reagent (Qiagen) with a final concentration of 5 nM at 50% confluence. The transfected cell were cultured with hypoxia (5% O_2_) or normoxia (21% O_2_) 24h after transfection. EGFR inhibitor Cetuximab was added 1h before hypoxia (5% O_2_) or normoxia (21% O_2_) culture. RNA and protein extraction with confirmed knock down efficiency were performed as previous described [31]. MTS assay (Promega, Madison, WI) was performed according to the protocol suggested by the manufacturer [32]. The percentage of cell growth was calculated based on 100% growth at 24 h after transfection.

### Transgenic mice HNSCC sample

The *Tgfbr1/Pten* 2cKO mice (K14-CreER^tam^; *Tgfbr1*^flox/flox^; *Pten*^flox/flox^), *Pten* cKO mice (K14-CreER^tam^; *Pten*^flox/flox^) and *Tgfbr1* cKO mice (K14-CreER^tam^; *Tgfbr1*^flox/flox^) HNSCC sample with counterpart control were kindly gifted by Dr. Ashok B. Kulkarni of National Institute of Dental Crainiofacial Research, NIH as previously described [27, 31, 43]. Cetuximab treated *Tgfbr1/Pten* 2cKO mice tissue were harvest from previous study [14].

### Human squamous cell carcinoma tissues array

Custome made tissue array T-12-412 and T12-412-TMA2 including 59 HNSCC, 39 mucosa and 13 dysplasia, 5 paired lymph node metastasis with clinicopathological data and follow up is made by Department of Oral Maxillofacial-Head Neck Oncology, School and Hospital of Stomatology, Wuhan University (PI: Zhi-Jun Sun) as previous described [45]. Human esophagus squamous cell carcinoma (ESCC) tissue array (HEso-seq172sur-02) including 93 ESCC and 79 paired esophagus mucosa were purchase from Shanghai Biochip (Shanghai, China). Clinicopathological data and 60-72 month follow up were providing in 85 ESCC.

### Inductive cisplatin based chemotherapy

12 human HNSCC sample with paired biopsy tissue were collected in Department of Oral Maxillofacial-Head Neck Oncology, School and Hospital of Stomatology, Wuhan University with confirm of Institute Reviewer Boad (Co-PI: Wen-Feng Zhang). All the patients receive same protocol of an open-label phase III trial multicenter double-blind, controlled prospective clinical trial [46]. Eligibility criteria included untreated stage III or IV with locally advanced resectable OSCC. Patients received two cycles of TPF induction chemotherapy (docetaxel 75 mg/m(2) on day 1, cisplatin 75 mg/m(2) on day 1, and fluorouracil 750 mg/m(2) on days 1 to 5) followed by radical surgery and postoperative radiotherapy versus up-front radical surgery and postoperative radiotherapy. Response to TPF chemotherapy were calculated according to RECIST (version 1.0) [47]. Immunohistochemistry of TFE3 on biopsy OSCC tissue and post-operative OSCC tissue were calculated blindly. A favorable response (pathologic complete response) was defined as absence of any tumor cells or presence of scattered foci of a few tumor cells with minimal residual disease with <10% viable tumor cells [46]. An unfavorable pathologic response was defined as the presence of >10% viable tumor cells in the resected specimen [46].

### Histology, immunohistochemistry and immunofluroscence

Histology and HE staining were performed as previous describe [27, 31, 43]. Immunohistochemistry for HIF-1α (Novus, 1:200), TFE3 (Novus, 1:400), PAI-1 (Proteintech, 1:100), EGFR (Cell signaling technology, 1:50) and Ki-67 (DAKO,1:400) was stained in serial-cut tissue array sections and sections of *Tgfbr1/Pten* 2cKO tongue SCC samples using an appropriate biotin- conjugated secondary antibody and a Vectastain ABC Elite kit (Vector Laboratories, Burlingame, CA, USA), as previously reported in protocols [27, 31, 43].

### Scoring system, hierarchical clustering and data visualization

All slices were scanned using an Aperio ScanScope CS scanner (Vista, CA, USA) with a background substrate for each slice, and quantified using Aperio Quantification software (Version 9.1) for membrane, nuclear, or pixel quantification [48]. An area of interest was selected either in the epithelial or the cancerous area for scanning and quantification. The histoscore of membrane and nuclear staining was calculated as a percentage of different positive cells using the formula (3+)×3+(2+)×2+(1+)×1. The histoscore of pixel quantification was calculated as the total intensity/total cell number [31]. The threshold for scanning of different positive cells was set according to the standard controls provided by Aperio [27, 31]. The expression scores were converted into scaled values centered on zero in Microsoft excel. Next, the hierarchical analysis was achieved using the Cluster 3.0 with average linkage based on Pearson's correlation coefficient [49]. Java TreeView 1.0.5 was used to visualize the results [50]. Finally, we arranged the clustered data and tissue samples on the horizontal axis and vertical axis respectively. Biomarkers with a close relationship are located next to each other.

### Western blot analysis

Cultured cells were lysed in T-PER (Pierce, Rockford, IL) containing a complete mini protease inhibitor cocktail and phosphate inhibitors (Roche, Branchburg, NJ). Tongue mucosa harvested from two individual *Tgfbr1*^flox/flox^/*Pten*^flox/flox^ mice and two *Tgfbr1/Pten* 2cKO mice, and five tumors harvested from *Tgfbr1/Pten* 2cKO mice, were used for Western blot analysis. Detailed procedures for immunoblotting were described as described previously [27, 31, 43].

### Statistical analysis

Data analyses were performed using Graph Pad Prism version 5.0 for Windows (Graph-Pad Software Inc, La Jolla, CA). One-way ANOVA followed by the post-Tukey or Bonferroni multiple comparison tests were used to analyze the differences in immunostaining and protein levels among each group. The Mann–Whitney *U* test was used to evaluate differences in the total tumor area of the mice treated with Cetuximab and of the untreated control group. Two-tailed Pearson statistics were used for correlated expression of TFE3 after confirmation of the sample with Gaussian distribution. Mean values ± SEM with a difference of *P* < 0.05 were considered statistically significant.

## SUPPLEMENTARY FIGURES


