# Supplementary material for: Hypoxia induces TFE3 expression in head and neck squamous cell carcinoma
Source: Oncotarget. 2016 Feb 10;7(10):11651–63. doi: 10.18632/oncotarget.7309 (PMC4905500; doi:10.18632/oncotarget.7309)
Supplement: Supplementary file 1 [file oncotarget-07-11651-s001.pdf]

SUPPLEMENTARY FIGURES

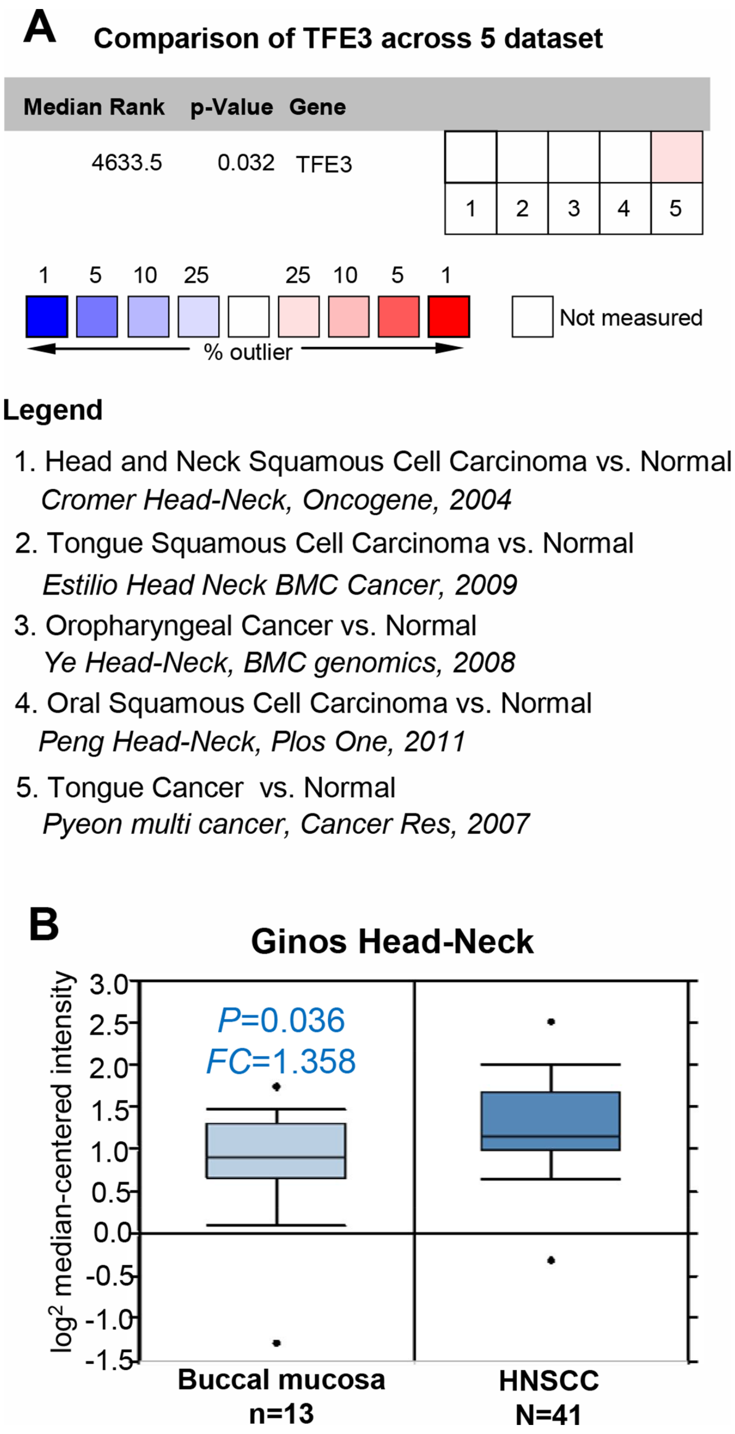

Supplementary Figure S1: DNA copy number and mRNA level of TFE3 in human head neck squamous cell carcinoma. **A.** TFE3 DNA copy number from Poage's dataset as indicated by $\log^2$ copy number unit of raw data. **B.** mRNA expression from Cromer's dataset as indicated by $\log^2$ median centered density of raw data. **C.** TFE3 mRNA expression in Acadesine resistant and Acadesine sensitivity HNSCC cells shown as box-whisker plot. **D.** Meta-analysis of 5 cohort of TFE3 expression shown as box-whisker plot (P=0.032). **E.** Meta analysis of gene expression profiling for TFE3 where the colored squares indicate the median rank for TFE3 across each analysis from 5 datasets in the ONCOMINE database (P=0.032).

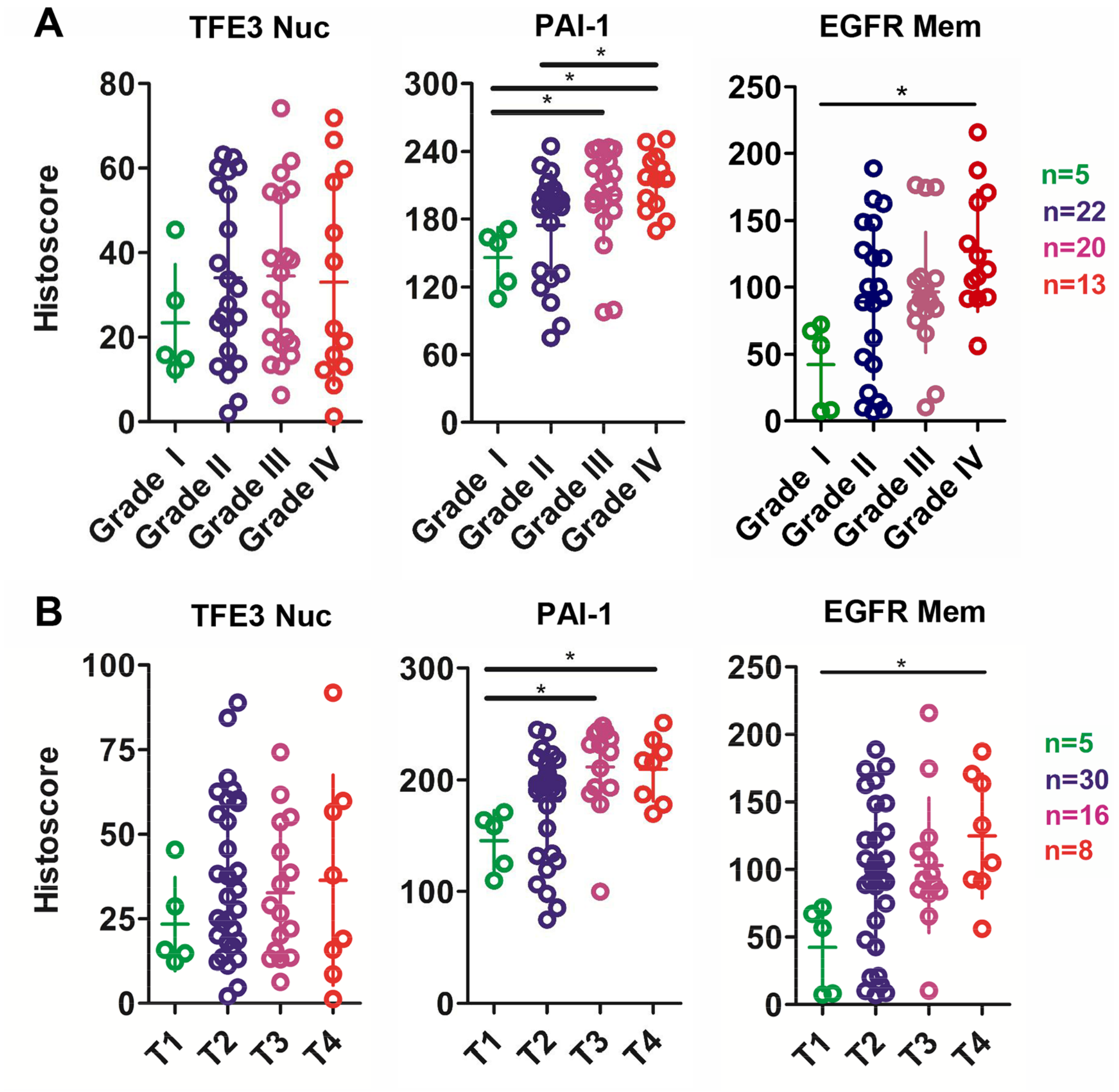

Supplementary Figure S2: Increase of TFE3 and PAI-1 in human HNSCC. A. TFE3 and PAI-1 immunostaining increased in high-grade HNSCC, but the difference was not statistically significant in TFE3, while PAI-1 expression significantly increased ($P < 0.05$) in poorly differentiated HNSCC samples compared with well-differentiated HNSCCs (Grade III vs. Grade I; Grade IV vs. Grade I; Grade IV vs. Grad II, $P<0.05$ respectively). **B.** TFE3 and PAI-1 immunostaining increased in large tumor size of HNSCC, but the difference was not statistically significant in TFE3, while PAI-1 expression increased in large-size HNSCC (T3 or T4) as compared with that in small-size HNSCC (T1, $P<0.05$). GraphPad Prism 5, one-way ANOVA with post Tukey statistic. *,$P<0.05$.

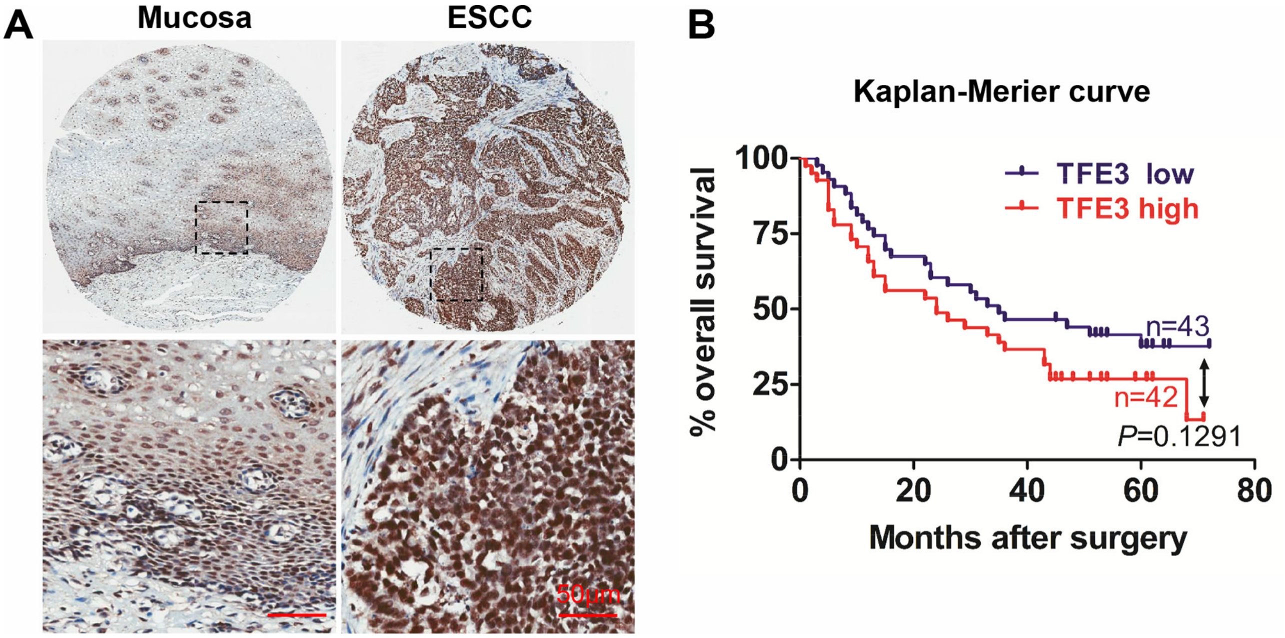

Supplementary Figure S3: Expression of TFE3 in esophagus squamous cell carcinoma. **A.** Representative immunohistochemistry staining of TFE3 in normal esophagus mucosa as well as in esophagus squamous cell carcinoma (ESCC) tissue (Scale bars =50um). **B.** Kaplan-Meier curve of TFE3 in esophagus squamous cell carcinoma indicated TFE3 high expression may distinct TFE3 low expression patient and suggest poorer prognosis, while the statistic is not significant (n=85, *P*=0.1291).

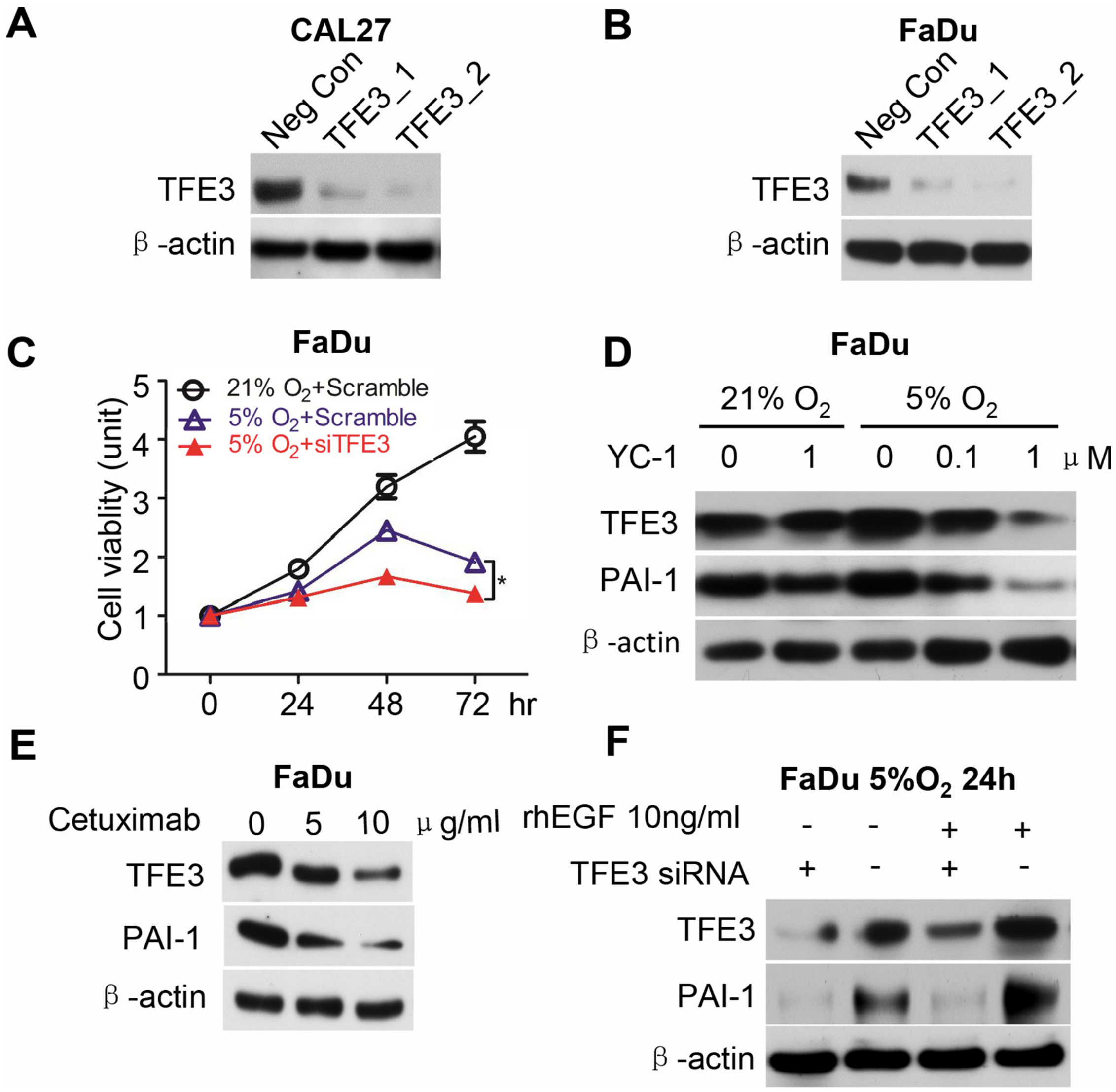

Supplementary Figure S4: Hypoxia increase TFE3 expression in FaDu. Protein level for TFE3 with two siRNA in the HNSCC cell line CAL27 **A.** and FaDu **B.** cell lines. **C.** TFE3 siRNA significantly reduces cell viability of FaDu in hypoxia culture condition at 48 and 72 h. **D.** Hypoxia inhibitor compound YC-1 treatment reduces effect on HIF-1α, TFE3, PAI-1 expression at a dose dependent manner in hypoxia culture condition, while it is less effect on FaDu in normoxia culture condition. **E.** Western blot analysis for TFE3 and PAI-1 in the FaDu HNSCC cell line after cetuximab treatment in a dose dependent manner. **F.** Western blot analysis for TFE3 and PAI-1 in the FaDu HNSCC cell line with recombination human EGF (10ng/ml) and TFE3 siRNA treated in hypoxia.

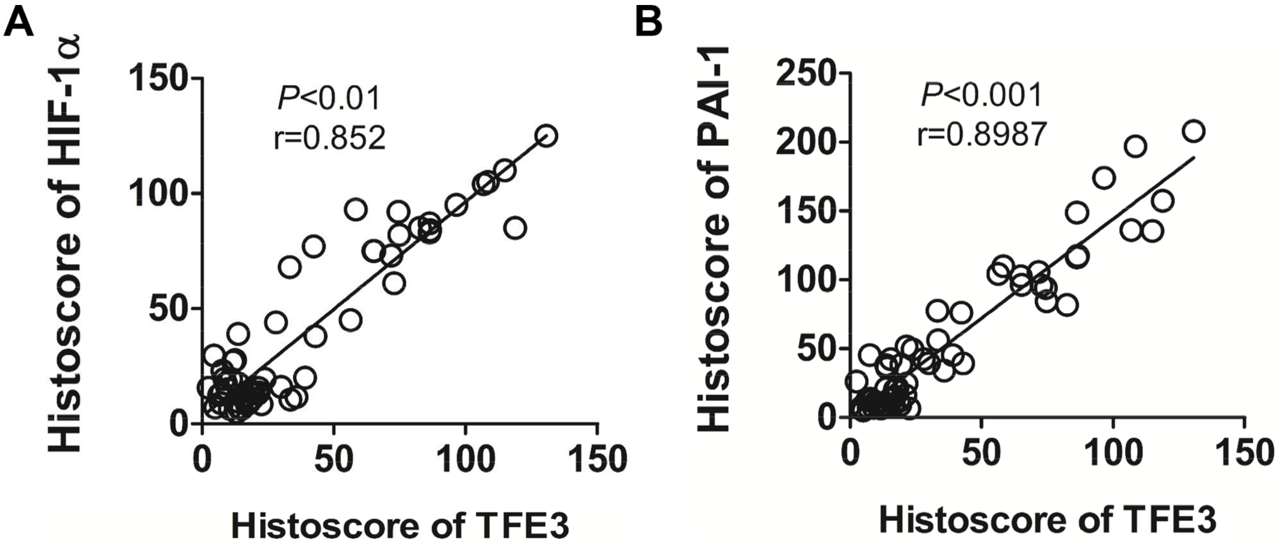

Supplementary Figure S5: TFE3 upregulation is correlated with HIF-1α and PAI-1 in *Tgfbr1/Pten* 2cKO mice bearing spontaneously developed HNSCC tumors. Quantification of immunohistochemical staining shows correlation of TFE3, HIF-1α and PAI-1 in *Tgfbr1/Pten* 2cKO mouse HNSCC, *Tgfbr1/Pten* 2cKO mouse mucosa and *Tgfbr1*$^{flox/flox}$/*Pten*$^{flox/flox}$ mucosa (n=5 mice in each group: wild type tongue, knock out tongue, knock out tongue cancer and 4 random high power view were selected of each slice).Data present as each view with statistic by Graph Pad Prism 5.

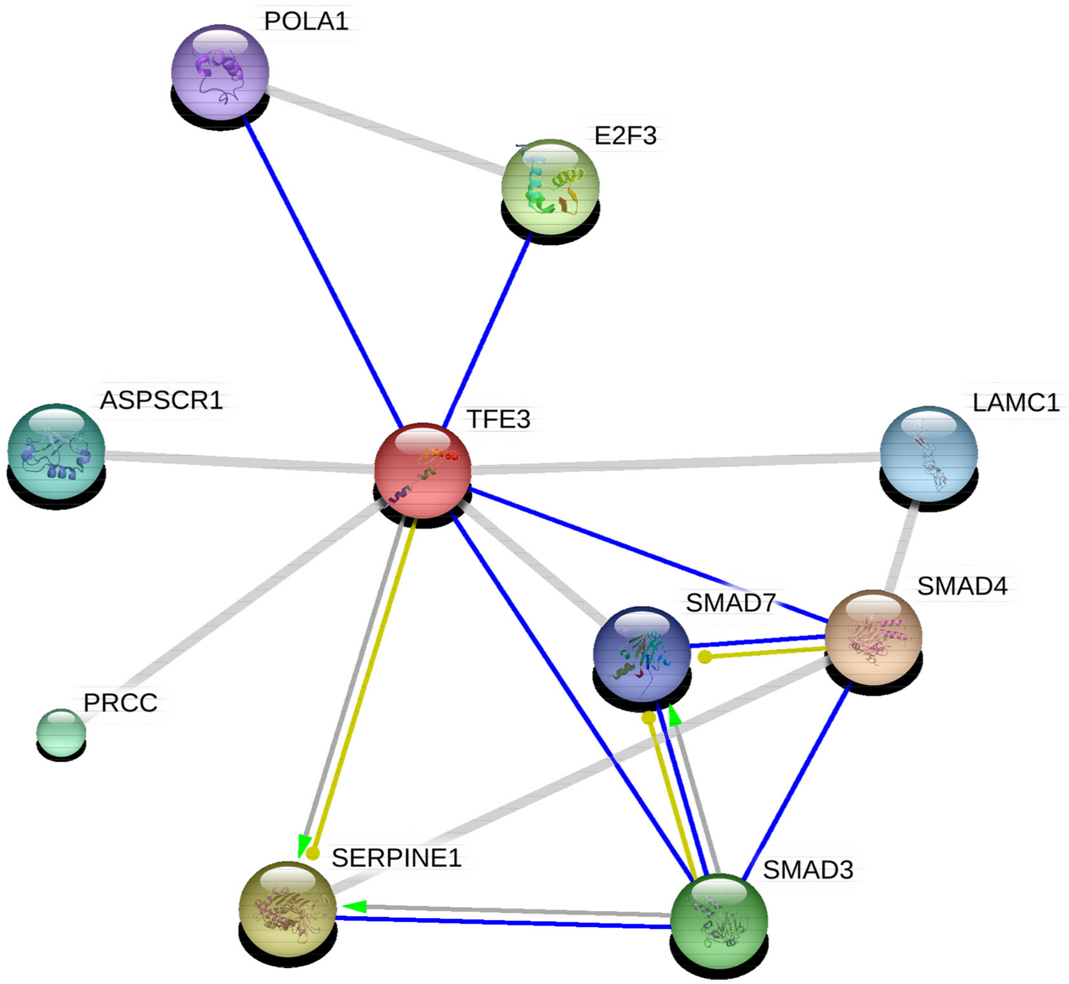

Supplementary Figure S6: Predict protein-protein interaction of TFE3. Computer predicts protein with direct protein-protein interaction with TFE3 analyzed by STRING database.
